# Kinetic analysis of the interactions between plant thioredoxin and target proteins

**DOI:** 10.3389/fpls.2013.00508

**Published:** 2013-12-18

**Authors:** Satoshi Hara, Toru Hisabori

**Affiliations:** ^1^Chemical Resources Laboratory, Tokyo Institute of TechnologyMidori-Ku, Yokohama, Japan; ^2^Core Research for Evolutional Science and Technology, Japan Science and Technology AgencyTokyo, Japan

**Keywords:** cysteine, protein–protein interaction, redox regulation, surface plasmon resonance, thioredoxin

## Abstract

Thioredoxin is a critical protein that mediates the transfer of reducing equivalents *in vivo* and regulates redox sensitive enzymes in several cases. In addition, thioredoxin provides reducing equivalents to oxidoreductases such as peroxiredoxin. Through a dithiol–disulfide exchange reaction, the reduced form of thioredoxin preferentially interacts with the oxidized forms of targets, which are immediately released after this reaction is complete. In order to more thoroughly characterize these interactions between thioredoxin and its target proteins, a mutant version of thioredoxin that lacked the second cysteine was synthesized and interactions were monitored by surface plasmon resonance. The binding rates of thioredoxin to its targets were very different depending on the use of reducing equivalents by the targets: the enzymes whose activity was controlled by reduction or oxidation of a cysteine pair(s) in the molecule and the enzymes that used reducing equivalents provided by thioredoxin for their catalysis. In addition, thioredoxin revealed a stronger preference for an oxidized target. These results explain the reason for selective association of thioredoxin with oxidized targets for reduction, whereas immediate dissociation from a reduced target when the dithiol–disulfide exchange reaction is complete.

## Introduction

Thioredoxin (Trx) is a small, ubiquitous protein that contains a pair of redox-sensitive cysteine residues within its catalytic domain. This domain comprises the highly conserved sequence motif Trp-Cys-Gly-Pro-Cys-[Lys/Arg] (Holmgren, 1985; Buchanan, 1991; Balmer et al., 2006). The cysteines allow Trx to reduce disulfide bonds on target proteins by catalyzing a dithiol–disulfide exchange reaction, which regulates target activity in several cases. On the basis of the reduction process mediated by Trx studies, the cysteine toward the N-terminal end of the Trx catalytic domain first attacks disulfide bond in a target protein to form an intermolecular disulfide complex, often described as a mixed disulfide intermediate complex (Brandes et al., 1993). Subsequently, the cysteine toward the C-terminal end of the Trx catalytic domain attacks this intermolecular disulfide bond, which results in an oxidized form of Trx and a reduced target.

Trx was first identified as a reducing equivalent donor for the deoxyribonucleotide reductase in *E. coli* (Laurent et al., 1964). In contrast to bacteria and animals, numerous isoforms of Trx have been identified in plants, although their specific roles are poorly understood (Balmer et al., 2006). Recent proteomics studies revealed that these Trx isoforms could react with a large number of candidate target proteins *in vitro* (Motohashi et al., 2001; Yano et al., 2001; Balmer et al., 2003; Yamazaki et al., 2004) and *in vivo* (Hall et al., 2010). For example, approximately 400 proteins have been described as potential targets of the plant Trx system (Montrichard et al., 2009), although only a small portion of these have been confirmed to be *bona fide* target proteins by biochemical analyses.

With regard to the target specificity and the Trx-mediated reaction process, a number of questions remain unanswered. For example, the mechanism of disulfide bonds recognition on the target proteins by Trx and, during the reaction process, the mechanism by which reduced form of Trx react with the oxidized form of the target protein and release it immediately after the dithiol–disulfide exchange reaction is completed. The target proteins have been categorized into two groups on the basis of their mode of interaction with Trx.

One group is the “switch” type proteins that are predominantly found within the chloroplast such as the chloroplast ATP synthase γ subunit (McKinney et al., 1979; Nalin and Mccarty, 1984), four Calvin cycle enzymes (glyceraldehyde 3-phosphate dehydrogenase, fructose 1,6-bis-phsophatase, sedoheptulose 1,7-bis-phosphatase, and phosphoribulokinase) (Jacquot et al., 1997), and malate dehydrogenase (MDH) (Scheibe and Anderson, 1981). The activities of the target proteins in this group are regulated through the oxidation/reduction of the Trx-targeted disulfide bond located within the molecule. Once reduced by Trx, these target proteins no longer require reducing equivalents provided by Trx.

The second group comprises the “catalytic” types such as peroxiredoxin (Dietz, 2003) and methionine sulfoxide reductase (MSR) (Tarrago et al., 2009). Because “catalytic” type target enzymes use reducing equivalents provided by Trx as an integral part of their catalytic process, they must repeatedly interact with Trx during the catalytic cycle of a target enzyme.

During the dithiol–disulfide exchange reaction, the reduced form of Trx associates with and reduces the oxidized form of the target protein, and then the interchange in redox conditions results in oxidized and reduced forms, respectively. Moreover, when a disulfide bond on the target protein is reduced by the reduced form of Trx, the target protein and Trx immediately dissociate for re-reduction of the oxidized form of Trx by Trx-reductase. This suggests that these proteins must drastically change their affinity for Trx before and after the dithiol–disulfide exchange reaction.

Till date, several attempts regarding the interactions between Trx and the target proteins have been reported. The key residues on the surface of a Trx-*f* molecule involved in target specificity were revealed by mutation analyses (Geck et al., 1996; Geck and Hartman, 2000). The altered S_0.5_ and V_max_ values for target enzyme activation caused by a Trx mutation indicated the importance of protein–protein interaction in addition to the dithiol–disulfide exchange reaction. In the case of the interaction between MSR and Trx from *E. coli*, the rate constants for the chemical reaction steps were determined by fluorescence stopped-flow measurements (Antoine et al., 2003; Olry et al., 2004). The authors concluded that the rate limiting step for regeneration of the reduced form of MSR by Trx was the dissociation of oxidized Trx from a reduced MSR complex.

Considering the Trx reduction reaction, there are two interaction modes between Trx and the target protein that depend on their redox states. One is the interaction between the reduced form of Trx and the oxidized form of target protein, and the other is the interaction between the oxidized form of Trx and the reduced form of target protein. Clarifying the difference between these interactions would be helpful for understanding Trx-mediated reduction mechanisms. However, conventional approaches are difficult to use for revealing the changes in affinity between Trx and the target proteins, in particular with regard to the redox states of Trx and its target proteins.

For characterization of these enigmatic but fundamental phenomena that occur within the Trx catalytic domain, we used surface plasmon resonance (SPR) to directly determine the association and dissociation rate constants of Trx to its target proteins. *Arabidopsis thaliana* cytosolic MDH and chloroplast peroxiredoxin Q (PrxQ) were used as “switch” and “catalytic” type target model proteins, respectively. Cytosolic MDH is a homodimeric enzyme that contains a Cys330–Cys330 intermolecular disulfide bond, and reduction of this disulfide bond by Trx has a strong effect on its activity (Hara et al., 2006). PrxQ is a chloroplast localized Trx-dependent peroxiredoxin, the activity of which is exhibited efficiently by the cytosolic Trx rather than the chloroplast Trx (Rouhier et al., 2004). Moreover, PrxQ is a monomeric protein that contains a Cys45–Cys50 intramolecular disulfide bond. Because MDH and PrxQ each have a disulfide bond, we selected them as target model proteins and Trx*h* as the reductant for this study.

## Materials and methods

### Proteins

Cloning and purification of *A. thaliana* cytosolic thioredoxin *h*1 (Trx*h*1) (AT3G51030), cytosolic MDH (AT1G04410), and chloroplast PrxQ (AT3G26060) were performed as described by Motohashi et al. (2001); Yamazaki et al. (2004); and Hara et al. (2006), respectively. roGFP1-iL was prepared as described by Lohman and Remington (2008) with minor modifications. This protein was used as the redox sensitive GFP derivative (roGFP) for this study. In brief, roGFP expressed in *E. coli* strain BL21(DE3) was purified by anion exchange chromatography using a DEAE-Toyopearl 650M and by hydrophobic interaction chromatography using a Butyl-Toyopearl 650 M.

A Trx*h*1 mutant with a C43S substitution (Trx*h*1_CS_) and a Trx*h*1 mutant with a C40S/C43S substitution (Trx*h*1_SS_) were prepared by the mega-primer method (Sarkar and Sommer, 1990). These were expressed and purified using the same procedures as for the wild-type Trx*h*1 with minor modifications. The Trx*h*1_CS_ and Trx*h*1_SS_ mutants were purified using a solution containing 0.5 mM DTT in all purification procedures to prevent unexpected disulfide bond formation.

The oxidized MDH was prepared by incubation with 50μM CuCl_2_ for 1 h at 30°C. Purified PrxQ and roGFP were obtained as oxidized forms. To obtain reduced PrxQ, oxidized PrxQ was incubated with 5 mM DTT and then dialyzed against well degassed H buffer (10 mM HEPES-NaOH, 150 mM NaCl, and 50μM EDTA, pH 7.4). The redox states of all proteins were confirmed by the AMS-labeling method with non-reducing SDS-PAGE (Motohashi et al., 2001). Protein concentrations were determined using a BCA assay with BSA as standard.

### SPR measurements

SPR measurements were carried out using a Biacore X system (GE Healthcare, Piscataway, NJ). All experiments were performed at 25°C with the indicated flow rates. All proteins were dialyzed against H buffer before the measurements. Ligand proteins were immobilized on a CM5 sensor chip at a flow rate of 5μl/min using amino coupling methods. After activation of the CM5 sensor chip with 0.2 M 1-ethyl-3-(3-dimethylaminopropyl) carbodiimide and 0.05M N-hydroxysulfosuccinimide (NHS) for 7 min, ligand proteins were diluted with 10 mM sodium acetate (pH 4.5) and immediately injected into the flow cell (Fc.2) onto the activated CM5 sensor chip using the manual injection mode. When sufficient amounts of ligand proteins were immobilized (equivalent to 800–900 RU signals on SPR), residual active NHS esters were blocked by injecting 1 M ethanolamine–HCl (pH 8.5) for 7 min. The reference flow cell (Fc.1) was treated in the same way without ligand proteins.

Experiments were performed after adding 0.005% Surfactant P20 (GE Healthcare) to H buffer. For repeat measurements, the surface of the sensor chip was regenerated with 5 mM DTT and 500 mM NaCl in H buffer for 2 min. The association and dissociation phase data (signal from Fc.2–signal from Fc.1) were used to determine kinetic parameters. A 1:1 binding model was used to obtain the rate constants for MDH. For PrxQ, data were fit to a double exponential model that had two individual pairs of association and dissociation rate constants. These analyses were done using a BIAevalution version 4.1 (GE Healthcare).

### Disulfide bond reduction on roGFP by Trx*h*1

Disulfide reduction on roGFP was fluorometrically evaluated using the excitation ratio of 395 nm/475 nm with emission at 510 nm. Oxidized roGFP (15μM) was incubated with 0.5 mM NADPH, 0.5 μM of NADPH-Trx reductase from *A. thaliana* (AtNTR), and 1 μM Trx*h*1 at 25°C for 30 min in H buffer. The excitation spectra were then measured using a FP-8500 spectrofluorometer (JASCO, Tokyo, Japan).

### Rate of disulfide bond reduction on MDH mediated by Trx*h*1

The extent of disulfide bond reduction was determined from the proportion of reduced forms in total MDH proteins. To reduce Trx*h*1, 0.5 mM NADPH, 0.5μM AtNTR, and 0.2–5μM Trx*h*1 were incubated at 25°C for 5 min in H buffer in advance. The reaction was initiated by adding 1μM oxidized MDH to this mixture, and at the indicated times, proteins in the aliquot were precipitated by adding 5% TCA (w/v). SDS-sample buffer containing 10 mM N-ethylmaleimide (NEM) was then added to these precipitants and proteins were separated by non-reducing SDS-PAGE. The band intensities (I_red_ and I_*ox*_ for reduced and oxidized proteins, respectively) were determined using a Scion Image software and the reduced proportion was calculated by the following equation: Reduced proportion (%) = I_red_/(I_red_ + I_ox_) × 100%.

### Disulfide bond reduction rate on PrxQ by Trx*h*1

The rate of disulfide bond reduction on PrxQ was estimated from the H_2_O_2_ reduction activity of PrxQ. This activity was determined using a coupling assay system that contained AtNTR and Trx. The decrease in absorbance at 340 nm due to NADPH oxidation was monitored (Motohashi et al., 2001). The reaction was initiated by adding 3μM Trx to a reaction mixture that contained 0.5mM NADPH, 1μM AtNTR, 0.5μM PrxQ, and 0.5 mM H_2_O_2_ in H buffer.

### Gel filtration chromatography

Gel filtration chromatography analysis was performed using a TSK-G2000SW_XL_ (Tosoh, Tokyo), which had been equilibrated with H buffer. Eluted proteins were monitored at 280 nm.

## Results

### Reduction of disulfide bond containing proteins by wild type Trx*h*1

We used the following three proteins for this study: MDH, PrxQ, and roGFP, each of which contained a disulfide bond. We first determined the rates of disulfide bond reduction in these proteins by wild type Trx*h*1 (Table 1). Although roGFP has a surface exposed disulfide bond, it is barely reduced by Trx*h*1 (Meyer et al., 2007). We previously reported that the MDH and PrxQ were reduced by Trx (Motohashi et al., 2001; Hara et al., 2006), although the reduction rates of these proteins were very different. This difference was probably caused not only by the different chemical reaction rates of disulfide bond reduction but also by the differences in protein–protein interactions between Trx and these target proteins.

**Table 1:** **Rate constants of target proteins interacting with Trx*h*1**.

**Method**	**Rates**	**roGFP**	**MDH**	**PrxQ**	
				**Rapid**	**Slow**
SPR^a^	k_on_ (M^−1^ s^−1^)	N.D.	247	7.97 × 10^4^	765
	koff (s^−1^)	N.D.	3.01 × 10^−4^	0.62	5.62 × 10^−4^
Reduction reaction	Reduction rate (s^−1^)	N.D.	6.5 × 10^−3^^b^	0.67^c^	

a *Association and dissociation rate constants were determined by using Trxh1_CS_ mutant*.

b *Reduction rate of MDH was determined from the reduction extent of MDH by wild type Trxh1 measured at the various reaction periods (for detail, see Materials and Methods)*.

c *Reduction rate of PrxQ was determined from the amount of NADPH consumption in the presence of AtNTR, wild type Trxh1, PrxQ and hydrogen peroxide. The amounts of PrxQ was compensated to be a rate limiting step (for detail, see Materials and Methods)*.

### Target preferences revealed by SPR measurements

We first attempted SPR measurement using a combination of wild type Trx*h*1 as a ligand and oxidized MDH as the analyte; however, no SPR signals could be detected using this combination. Thus, we used the Trx*h*1_CS_ mutant as the ligand in subsequent experiments. The Trx*h*1_CS_ lacks the second cysteine in its catalytic domain and cannot catalyze the complete exchange of disulfide bond pairs with the target proteins. Thus, this appeared to be a good experimental model protein to capture a snapshot of the dithiol–disulfide exchange reaction mediated by Trx. Although this mutant cannot be used to imitate the actual affinity due to the formation of a mixed disulfide bond, this mutant exhibited association and dissociation activity in our SPR measurements. Therefore, we describe the data obtained in this study as apparent affinities for Trx and the target proteins.

**Figure 1:**
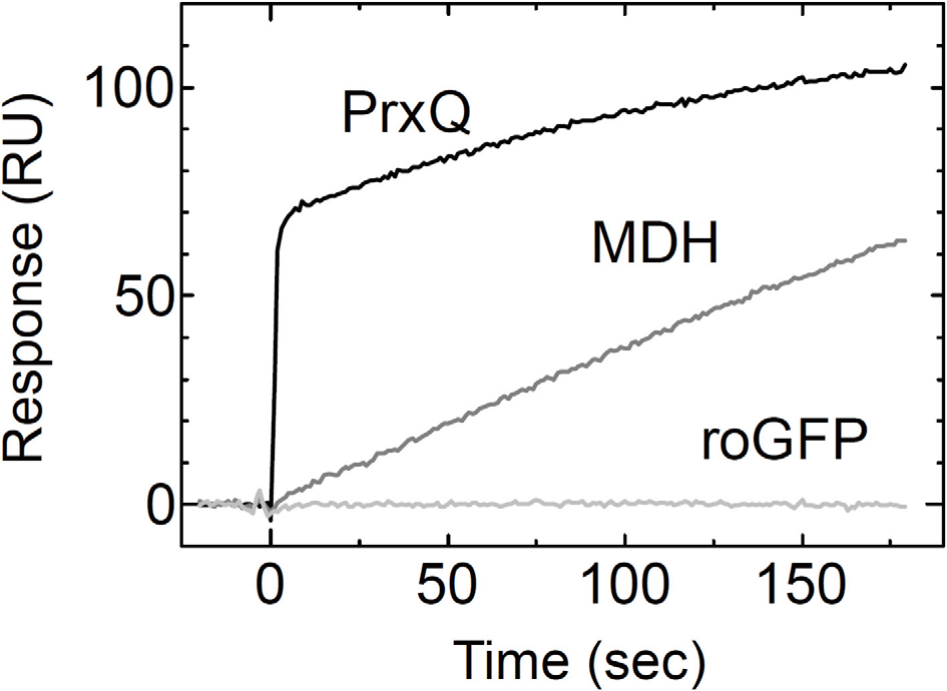
**Associations of model proteins with the Trx*h*1_CS_**. PrxQ, MDH, or roGFP at 5μM were injected onto the Trx*h*1_CS_ immobilized on a CM5 sensor chip. Experiments were performed at a flow rate of 30μl/min for PrxQ and 10μl/min for MDH and roGFP.

Using the Trx*h*1_CS_, we attempted to observe the dissociations and associations between Trx and three proteins, roGFP, PrxQ, and MDH, as SPR signals. These proteins have redox-sensitive disulfide bonds in their oxidized states. Thus, the resulting SPR signals should have been due to the formation of mixed disulfide intermediate complexes. When these proteins were injected into the Trx*h*1_CS_ immobilized on a CM5 sensor chip for SPR measurements, the sensorgrams for the association phase were very different (Figure 1). When roGFP with a surface-based Trx irreducible disulfide bond was used as the analyte, the SPR signals were barely detectable. This suggested that the Trx*h*1_CS_ did not associate non-specifically with this protein, even if the protein contains disulfide bond(s) on its surface. In contrast, PrxQ and MDH provided more significant SPR signals. These sensorgrams clearly revealed that the binding rate of PrxQ to the Trx*h*1_CS_ was much faster than that of MDH. These results were in accordance with the significant differences in their reduction rates of the disulfide bonds located on MDH (6.5 × 10^−3^ s^−1^) and PrxQ (0.67 s^−1^) by the wild type Trx*h*1 (Table 1).

### Kinetic analysis of the interaction between MDH and Trx

**Figure 2:**
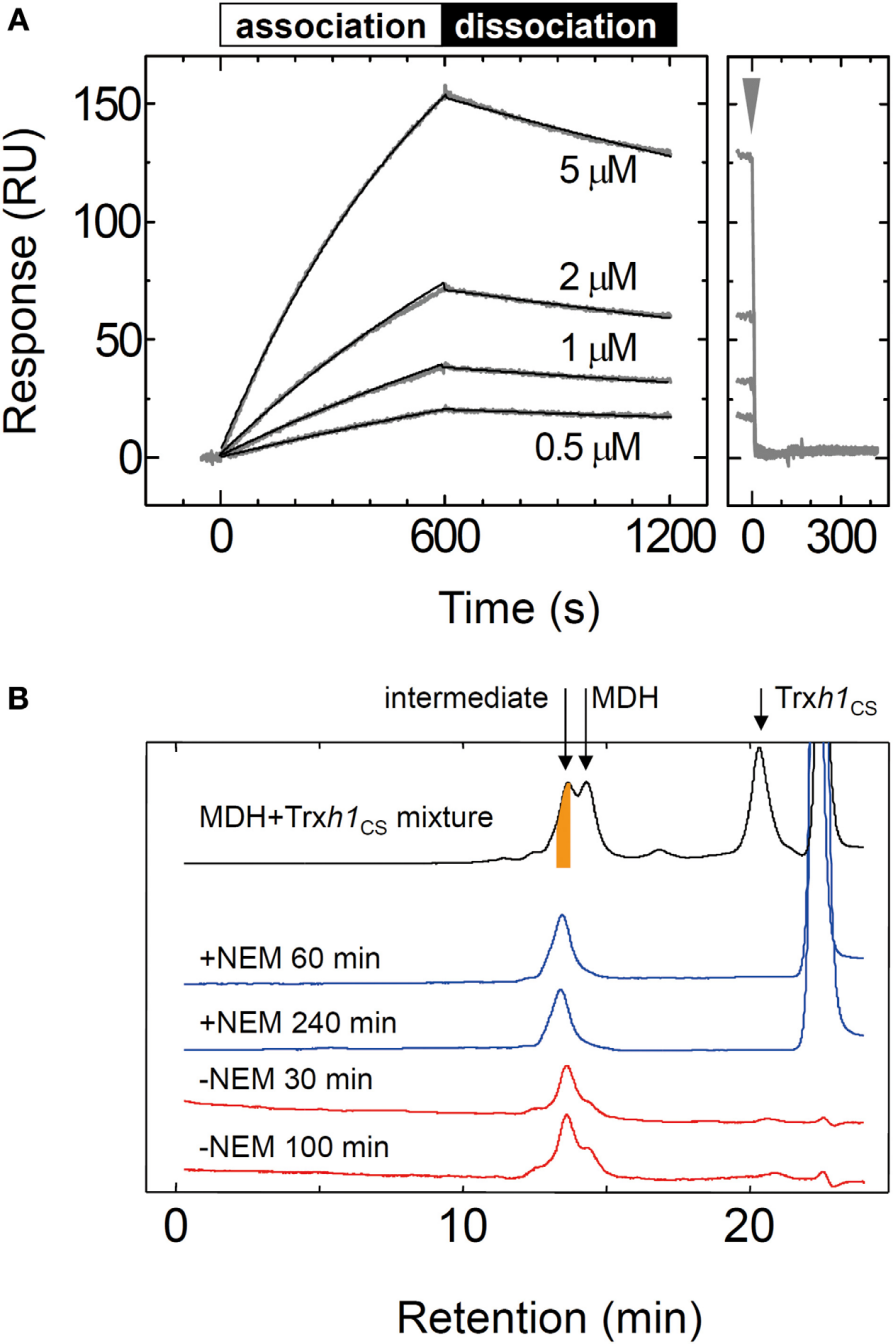
**Interaction between the oxidized form of the MDH and Trx*h*1_CS_ proteins. (A)** To investigate the association and dissociation phases, the indicated concentrations of MDH were injected for 600 s, followed by injection by buffer for additional 600s (left panel) and the SPR signals were recorded. Fitted curves are indicated by the black lines on the SPR sensorgram. The association and dissociation phases are indicated by bars on the sensorgram. The arrow indicates the time when 5 mM DTT was injected (right panel). **(B)** Mixed disulfide intermediate complexes formed by the Trx*h*1_CS_ and oxidized MDH were separated by gel filtration chromatography (TSK-G2000SW_XL_) (black trace). The intermediate complexes (orange colored portion) were fractionated and then applied to the same column chromatography after incubation for the indicated period in the presence (blue trace) or the absence (red trace) of 10 mM NEM.

To thoroughly investigate the interaction between the MDH and Trx*h*1_CS_, we determined the rate constants for association and dissociation with the Trx*h*1_CS_ using SPR (Figure 2A). These data were fit to a 1:1 binding model, from which we obtained the association rate constant, k_on_ = 2.5 × 10^2^ M^−1^ s^−1^, and dissociation rate constant, k_off_ = 3.0 × 10^−4^ s^−1^ (Table 1). MDH dissociated from the immobilized Trx*h*1_CS_ during these measurements, although the increase in the SPR signals that were observed indicated the formation of an irreversible disulfide bond.

We then used gel filtration chromatography to determine the cause for MDH dissociation from the Trx*h*1_CS_ mutant. To form a mixed disulfide intermediate complex between the Trx*h*1_CS_ and MDH, 44μM of the oxidized form of MDH was incubated with 160μM Trx*h*1_CS_ for 30 min at 25°C. This protein mixture was then applied to the TSK-G2000SW_XL_ column and the desired peak with a retention time of 13.5 min that contained the mixed disulfide intermediate complexes was fractionated (orange colored portion in Figure 2B). The thiol alkylation reagent NEM was immediately added to this collected fraction and chromatography was repeated (Figure 2B).

When NEM was added to this fraction, the mixed disulfide intermediate complexes were maintained during the second round of chromatography and the protein peak on the second chromatography run was observed at the same retention time. In contrast, the intermediate protein complexes readily dissociated and MDH dimers were observed when the second round of chromatography was performed without NEM treatment in advance. These results clearly indicated that the dissociation of the mixed disulfide intermediate proteins occurred following nucleophilic attack on the disulfide bond by the thiol residue on the target protein, presumably observed as dissociation on the SPR sensorgram.

### Kinetic analysis of the interaction between PrxQ and Trx

**Figure 3:**
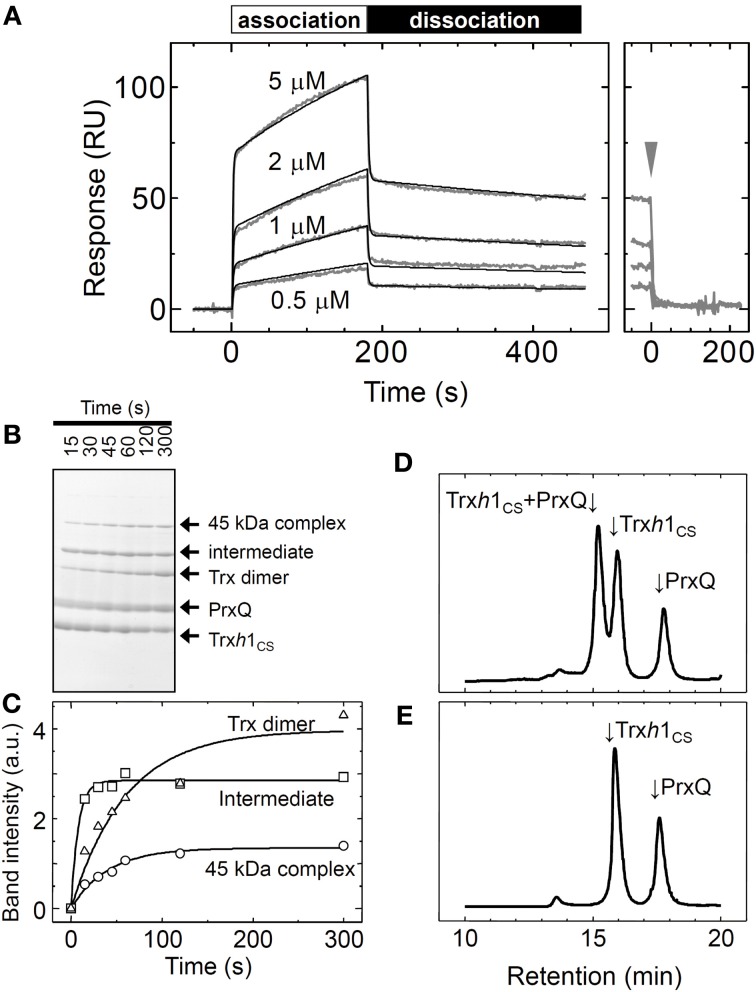
**Interaction between the oxidized form of the PrxQ and Trx*h*1_CS_ proteins. (A)** Same experiment as presented in Figure 2A, except using PrxQ as the analyte. **(B)** Trx*h*1_CS_ (50μM) and PrxQ (50μM) were incubated for the periods indicated. The reaction was then quenched with 5% TCA (w/v). Proteins were separated by non-reducing SDS-PAGE. **(C)** Band intensities of the mixed disulfide intermediate complexes (intermediate), 45 kDa complexes, and the Trx*h*1_CS_ dimers (Trx dimer) obtained from SDS-PAGE presented in panel **(B)** were determined using a Scion image software and plotted. a.u.; arbitrary units. **(D)** Trx*h*1_CS_ (50μM) and oxidized PrxQ (50μM) were incubated for 3 min at room temperature, and the complexes that formed were then incubated with 10 mM NEM. The protein mixture was then loaded onto G2000SW_XL_ and separated. **(E)** Same experiment as in **(D)** except for incubation in the absence of NEM.

The sensorgram for the interaction between the Trx*h*1_CS_ and PrxQ is presented in Figure 3A. Both association and dissociation phases revealed a biphasic interaction. Thus, these data were fit to a double-exponential equation, from which we obtained two rate constant pairs (Table 1). To identify the protein complexes involved in these observed biphasic reactions, 50μM Trx*h*1_CS_ and 50μM PrxQ were incubated for the periods indicated (Figures 3B,C). Mixed disulfide intermediate complex formation reactions were then quenched by adding 5% TCA (w/v) and proteins were separated by non-reducing SDS-PAGE.

From N-terminal amino acid sequence analysis, we confirmed that the two bands labeled “45kDa” and “intermediate” were composed of the Trx*h*1_CS_ and PrxQ through intermolecular disulfide bonds (Figure 3B). Although the stoichiometry for these proteins in this complex remains to be determined, the 45 kDa protein could be composed of one Trx*h*1_CS_ and two PrxQ molecules linked through the intermolecular disulfide bonds. The apparent formation rate for the one-to-one mixed disulfide intermediate complex between the Trx*h*1_CS_ and PrxQ was sufficiently fast and formation of this complex reached a plateau in less than 15 s.

The Trx*h*1_CS_ dimer formation and 45 kDa band formation occurred after this mixed disulfide intermediate complex was formed (Figure 3C). Because the SPR sensorgram additionally revealed two binding phases, faster and slower, we concluded that the faster phase most possibly corresponded to the formation of the mixed disulfide intermediate complex and the latter corresponded to the formation of the 45 kDa protein complex (Figure 3C).

We then used gel filtration chromatography to determine whether the rapid dissociation rate (k_off−rapid_ = 0.62 s^−1^) observed with SPR measurements corresponded to the release of the target protein from the mixed disulfide intermediate complex. For this purpose, 50μM Trx*h*1_CS_ and 50μM PrxQ were incubated for 3 min at 25°C to prepare mixed disulfide intermediate complexes, and then this protein mixture was treated with 10 mM NEM. Proteins were then loaded onto the TSK-G2000SW_XL_. Mixed disulfide intermediate complexes were only observed when prior treatment with NEM was used (Figures 3D,E). This indicated that mixed disulfide intermediate complexes comprising the Trx*h*1_CS_ and PrxQ would completely dissociate into monomeric proteins during gel filtration chromatography, similar to the MDH/Trx*h*1_CS_ complexes when the second cysteines on PrxQ were functional.

These data suggested that the rate constants obtained for the rapid phase presented in Table 1 corresponded to the formation and reverse dissociation reactions of mixed disulfide intermediate complexes.

### Redox state of the target proteins is required for efficient interaction

**Figure 4:**
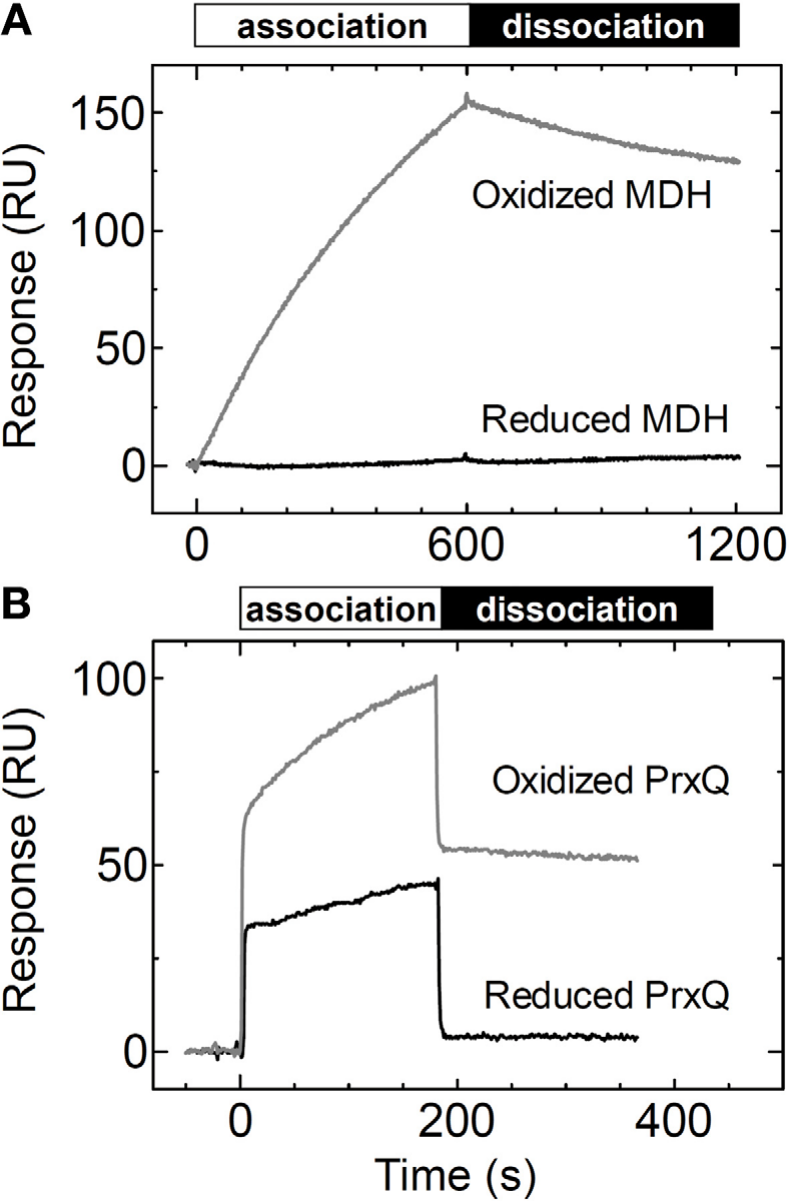
**Changes in affinity based on the redox states of the target proteins. (A)** Reduced or oxidized MDH (5μM) was injected onto the Trx*h*1_CS_ immobilized on a sensor chip and SPR signals were recorded. **(B)** Reduced or oxidized PrxQ (5μM) was injected onto the Trx*h*1_CS_ immobilized on a sensor chip and SPR signals were recorded.

Trx obviously recognizes the redox state of the target proteins *in vivo* to efficiently reduce them. We aimed to determine whether the redox state of the target protein affects the affinity between the target protein and Trx. We measured the binding of MDH and PrxQ as analytes to the Trx*h*1_CS_ by SPR. When the reduced form of MDH was injected onto the Trx*h*1_CS_ immobilized on a CM5 sensor chip, no SPR signals were detected, although sufficient binding could be detected when oxidized MDH was used (Figure 4A).

In contrast, the reduced form of PrxQ could partially associate with the Trx*h*1_CS_, although the sensorgram clearly revealed a quantitative difference as compared to the oxidized form of PrxQ (Figure 4B). In fact, the reduced form of PrxQ exhibited immediate association and dissociation with the Trx*h*1_CS_ in a manner similar to that of the oxidized form of PrxQ. However, the SPR signals with the reduced form of PrxQ disappeared immediately when buffer was injected onto the sensor chip, which indicated that the affinity between the Trx*h*1_CS_ and the reduced form of PrxQ was very weak.

### Significance of protein–protein interactions

**Figure 5:**
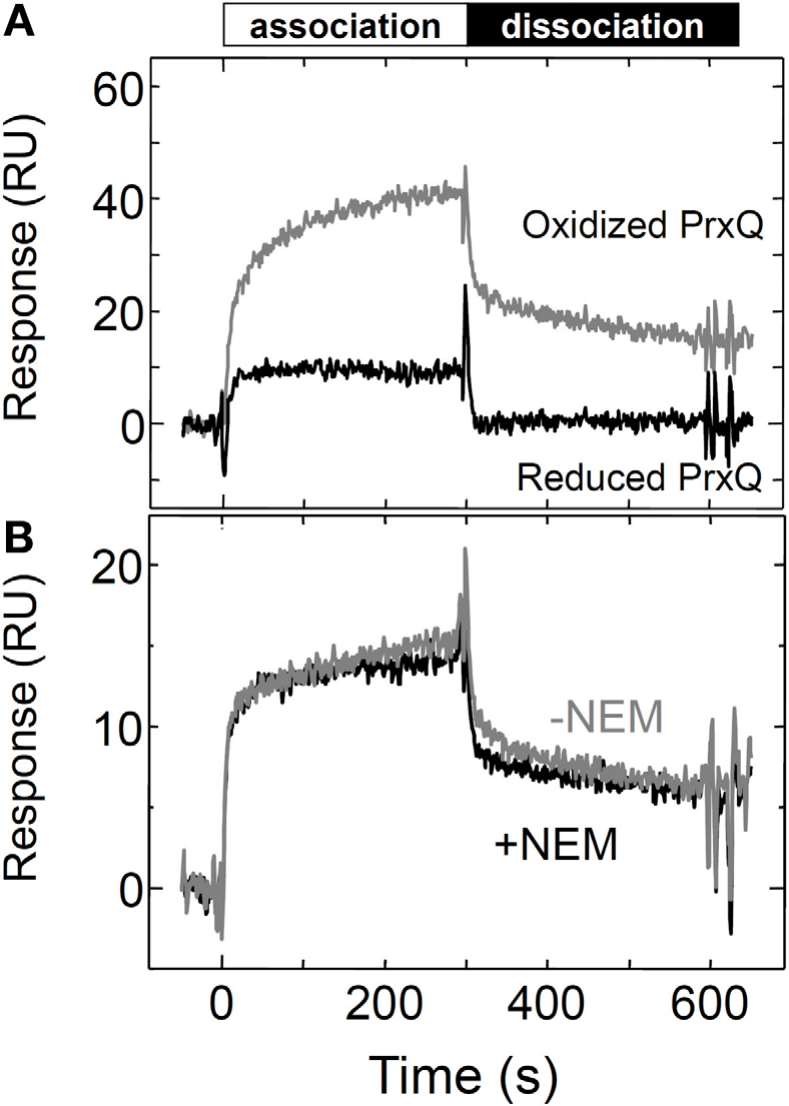
**Interactions between the Trx*h*1_SS_ and the reduced and oxidized forms of PrxQ. (A)** Reduced or oxidized PrxQ (5μM) was injected onto the Trx*h*1_SS_ immobilized on a sensor chip and SPR signals were recorded. **(B)** The oxidized form of PrxQ (2μM) was injected onto Trx*h*1_SS_ immobilized on a CM5 sensor chip before (gray line) and after (black line) thiol alkylation. Thiol alkylation was accomplished by injecting 10 mM NEM for 10 min onto the sensor chip.

Finally, to investigate the contribution of the surface properties of Trx for its interaction with the target proteins, we examined the association of Trx*h*1_SS_ with PrxQ by substitution of both the cysteines with serine. As presented in Figure 5A, the Trx*h*1_SS_ interacted with the oxidized form of PrxQ. This interaction between the Trx*h*1_SS_ and the oxidized form of PrxQ was not affected by alkylation of the additional cysteine at position 11 of the immobilized Trx*h*1_SS_ using 10 mM NEM (Figure 5B). This suggested that the observed binding of the oxidized form of PrxQ to the immobilized Trx*h*1_SS_ was due to a protein–protein interaction without intermolecular disulfide bond formation. The observed binding rate was slower than that for the Trx*h*1_CS_.

## Discussion

Several studies attempted to evaluate the affinities of Trx to its target proteins or the reduction efficiencies of the target proteins by determining K_m_ or k_cat_/K_m_ values obtained from the results of complete reduction of the target proteins using Trx (Collin et al., 2003; Perez-Perez et al., 2009; Maeda et al., 2010). However, a rather enigmatic behavior of Trx remained unresolved; the reduced form of Trx preferentially interacts with the oxidized form of the target protein and the oxidized form of Trx must release the reduced form of the target in order to ensure an efficient reduction cycle. To address this issue, we applied the direct binding measurements to detect the associations and dissociations of Trx with its target proteins.

For this purpose, MDH, PrxQ, and roGFP were selected as model target proteins. Cytosolic MDH is reportedly a target protein for Trx*h*1 (Hara et al., 2006) whose activity is regulated by its reduction/oxidation. Therefore, we defined this type of target protein as a “switch” type target for Trx. In contrast, PrxQ uses reducing equivalents provided by Trx for catalysis; thus, we defined it as a “catalytic” type target. Although PrxQ is localized in the chloroplasts of higher plants, cytosolic Trx can be an efficient reductant for this peroxiredoxin *in vitro*, as previously described (Rouhier et al., 2004). In addition, the reduction rate of PrxQ by Trx*h*1 (0.67 s^−1^) obtained in this study was comparable to or higher than the reduction rate of PrxQ reduced by chloroplast type Trx (Collin et al., 2004; Perez-Perez et al., 2009). In this study, we selected a combination of PrxQ and Trx*h*1 for our binding study because PrxQ has only one pair of cysteines in its monomeric molecule and its reaction mode is the simplest among peroxiredoxins.

When the wild type Trx*h*1 was used as a ligand, no SPR signals were detected with MDH as the analyte. Based on the reaction mechanism of Trx-mediated reduction of a target disulfide bond, the target protein should be immediately released from Trx when the reduction reaction is complete. This implies that the number of bound target proteins on immobilized Trx should be statistically limited by the duration of the reaction period from the formation of a mixed disulfide bond to its reduction. The time during which the target protein remains on immobilized Trx may be too short to allow for detecting the bound molecules on the gold surface of an SPR sensor chip. In addition, when the reduction reaction is completed by the dithiol–disulfide exchange reaction, immobilized Trx*h*1 adopts its oxidized form and can no longer reduce the target protein because there is no way to simultaneously reduce immobilized Trx*h*1 alone. Thus, a direct analysis of the binding affinity of Trx to its target protein is difficult.

Therefore, we used the immobilized Trx*h*1_CS_, which had been used for Trx affinity chromatography (Yamazaki et al., 2004), as the ligand in our SPR measurements. Because the Trx*h*1_CS_ could efficiently capture the target protein molecules through intermolecular disulfide bond formation, we expected that this mutant Trx could be used for observing the formation of a mixed disulfide intermediate complex, which is the initial contact of Trx and its target protein during the entirety of a reduction reaction. In contrast, a Trx*h*1_SC_ mutant, a Trx*h*1 mutant with a C40S substitution, was not used for these measurements because this mutant could not capture any specific target proteins when used for Trx affinity chromatography (Yamazaki et al., 2004).

As presented in Figure 1, SPR signals for the association of the analyte protein with the ligand were successfully obtained by using the Trx*h*1_CS_ as the ligand. In contrast, the Trx*h*1_CS_ did not associate with roGFP, which contains a disulfide bond that cannot be reduced by Trx. This indicated that the immobilized Trx*h*1_CS_ maintained specificity for the target proteins. In addition, the remarkable differences between the binding time courses for MDH and PrxQ (Figure 1) suggested that the method applied in this study was useful for our purposes.

Because the fast dissociation rate of the analyte from the mixed disulfide intermediate complex by the reverse reaction (k_off−rapid_ = 0.62 s^−1^) appear to pose difficulty in accurate determination of the association rate constant when performing SPR analysis using PrxQ, we concluded that the association rate constant for the formation of the mixed disulfide intermediate complex was in the order of 10^4^ M^−1^ s^−1^. In addition, the association rate constant for PrxQ was higher than that for MDH by two orders of magnitude. These results were comparable to the reported differences in the reduction rates of PrxQ and MDH by the wild type Trx*h*1; 0.67 s^−1^ and 6.5 × 10^−3^ s^−1^, respectively (Table 1).

Why did Trx (Trx*h*1_CS_) that mimics the reduced form of Trx interact with the oxidized form of MDH but not with the reduced form as presented in Figure 4A? A significant conformational change during the transition between the oxidized form and the reduced form of an MDH dimer has been suggested by size exclusion chromatography and analytical ultracentrifugation (Hara et al., 2006). This large conformational change must be critical for forming an intermolecular disulfide bond between Cys330–Cys330. In addition, the structure or the molecular surface of MDH for its interaction with Trx may only be exposed in its oxidized form but not its reduced form because on the basis of its reported crystal structure, the two Cys330 residues are located at the opposite sides in an MDH dimer molecule. This may be the reason for interaction of the Trx*h*1_CS_ with the oxidized form of MDH but not with the reduced form as presented in Figure 4A. In addition, a redox-dependent large conformational change has been reported for the other switch type target protein, HSP33. For HSP33, which is known to be a redox-dependent chaperone that contains four redox responsive cysteines, the domain that contains Trx-targeted disulfide bonds is largely unfolded in the oxidized form, although the reduced form of HSP33 is completely folded and coordinates zinc in this domain (Ilbert et al., 2007).

In contrast, the Trx*h*1_CS_ could bind to the reduced form of PrxQ as presented in Figure 4B. On the basis of the reported structures of the reduced and oxidized forms of PrxQ from *Aeropyrum pernix* (*Ap*PrxQ), a redox-dependent conformational change was clearly observed at the region that contained two active cysteines (Perkins et al., 2012). In contrast, molecular modeling for BCP1, the homolog of PrxQ in *Sulfolobus solfataricus*, and its reductase, *Ss*PDO (Limauro et al., 2010), revealed that the stable regions such as the end of β3 and α3 were involved in the molecular interaction, although these regions do not change their conformations in *Ap*PrxQ in a redox-dependent manner. These reports suggest that a certain stable region on a PrxQ protein can interact with its partner protein irrespective of their redox states. The reduced form of PrxQ may have a similar interaction region on its molecular surface, and it may have caused the relatively weak association between the Trx*h*1_CS_ and the reduced form of PrxQ in our SPR experiments.

In this study, we conclusively confirmed that the redox state of the target protein was the significant determinant for its affinity to Trx. As presented in Figure 4, MDH and PrxQ altered their affinities for Trx*h*1. As previously noted, the Trx*h*1_CS_ cannot associate with the reduced form of MDH. The amount of the reduced form of PrxQ that associated with Trx was definitely less than that of its oxidized form. These results strongly suggest that the interaction between Trx and an oxidized target protein exhibits a higher affinity than that between Trx and the reduced form, a prerequisite for efficient association and subsequent dissociation.

In addition, the Trx*h*1_SS_ exhibited a preference for the oxidized target protein, even though its active domain lacks two cysteines (Figure 5). This indicates that the protein–protein interaction defined by the molecular surfaces of Trx and its target proteins is an important determinant for the redox-dependent selectivity of Trx, in addition to the cysteines in the catalytic domain of Trx and the rate of formation of the mixed disulfide intermediate complex. Similarly, the interaction between a Trx mutant that lacked two cysteines and its target ATP synthase complex was previously reported (Stumpp et al., 1999).

Considering together, these data reveal that the efficient reduction of the target proteins by Trx is accomplished by a remarkable change in the affinity between Trx and its target protein before and after the dithiol–disulfide exchange reaction. This selectivity is defined by these protein–protein interactions. The key molecular determinants of Trx that confer these interesting properties need to be elucidated by structural analysis of the Trx–target protein co-complexes.

### Conflict of interest statement

The authors declare that the research was conducted in the absence of any commercial or financial relationships that could be construed as a potential conflict of interest.

## References

[B1] Antoine M. Boschi-Muller S. Branlant G. (2003). Kinetic characterization of the chemical steps involved in the catalytic mechanism of methionine sulfoxide reductase A from *Neisseria meningitidis*. J. Biol. Chem. 278, 45352–45357 10.1074/jbc.M307471200 12954610

[B2] Balmer Y. Koller A. Del Val G. Manieri W. Schurmann P. Buchanan B. B. (2003). Proteomics gives insight into the regulatory function of chloroplast thioredoxins. Proc. Natl. Acad. Sci. U.S.A. 100, 370–375 10.1073/pnas.232703799 12509500 PMC140980

[B3] Balmer Y. Vensel W. H. Hurkman W. J. Buchanan B. B. (2006). Thioredoxin target proteins in chloroplast thylakoid membranes. Antioxid. Redox Signal. 8, 1829–1834 10.1089/ars.2006.8.1829 16987035

[B4] Brandes H. K. Larimer F. W. Geck M. K. Stringer C. D. Schurmann P. Hartman F. C. (1993). Direct identification of the primary nucleophile of thioredoxin f. J. Biol. Chem. 268, 18411–18414 8395501

[B5] Buchanan B. B. (1991). Regulation of CO2 assimilation in oxygenic photosynthesis: the ferredoxin/thioredoxin system. Perspective on its discovery, present status, and future development. Arch. Biochem. Biophys. 288, 1–9 10.1016/0003-9861(91)90157-E 1910303

[B6] Collin V. Issakidis-Bourguet E. Marchand C. Hirasawa M. Lancelin J. M. Knaff D. B. (2003). The *Arabidopsis* plastidial thioredoxins: new functions and new insights into specificity. J. Biol. Chem. 278, 23747–23752 10.1074/jbc.M302077200 12707279

[B7] Collin V. Lamkemeyer P. Miginiac-Maslow M. Hirasawa M. Knaff D. B. Dietz K. J. (2004). Characterization of plastidial thioredoxins from *Arabidopsis* belonging to the new y-type. Plant Physiol. 136, 4088–4095 10.1104/pp.104.052233 15531707 PMC535839

[B8] Dietz K. J. (2003). Plant peroxiredoxins. Annu. Rev. Plant Physiol. Plant Mol. Biol. 54, 93–107 10.1146/annurev.arplant.54.031902.134934 14502986

[B9] Geck M. K. Hartman F. C. (2000). Kinetic and mutational analyses of the regulation of phosphoribulokinase by thioredoxins. J. Biol. Chem. 275, 18034–18039 10.1074/jbc.M001936200 10751409

[B10] Geck M. K. Larimer F. W. Hartman F. C. (1996). Identification of residues of spinach thioredoxin f that influence interactions with target enzymes. J. Biol. Chem. 271, 24736–24740 10.1074/jbc.271.40.24736 8798742

[B11] Hall M. Mata-Cabana A. Akerlund H. E. Florencio F. J. Schroder W. P. Lindahl M. (2010). Thioredoxin targets of the plant chloroplast lumen and their implications for plastid function. Proteomics 10, 987–1001 10.1002/pmic.200900654 20049866

[B12] Hara S. Motohashi K. Arisaka F. Romano P. G. Hosoya-Matsuda N. Kikuchi N. (2006). Thioredoxin-*h*1 reduces and reactivates the oxidized cytosolic malate dehydrogenase dimer in higher plants. J. Biol. Chem. 281, 32065–32071 10.1074/jbc.M605784200 16945919

[B13] Holmgren A. (1985). Thioredoxin. Annu. Rev. Biochem. 54, 237–271 10.1146/annurev.bi.54.070185.001321 3896121

[B14] Ilbert M. Horst J. Ahrens S. Winter J. Graf P. C. Lilie H. (2007). The redox-switch domain of Hsp33 functions as dual stress sensor. Nat. Struct. Mol. Biol. 14, 556–563 10.1038/nsmb1244 17515905 PMC2782886

[B15] Jacquot J. P. Lancelin J. M. Meyer Y. (1997). Thioredoxins: structure and function in plant cells. New Phytol. 136, 543–570 10.1046/j.1469-8137.1997.00784.x 33863109

[B16] Laurent T. C. Moore E. C. Reichard P. (1964). Enzymatic synthesis of deoxyribonucleotides. IV. Isolation and characterization of thioredoxin, the hydrogen donor from *Escherichia coli* B. J. Biol. Chem. 239, 3436–3444 14245400

[B17] Limauro D. D'Ambrosio K. Langella E. de Simone G. Galdi I. Pedone C. (2010). Exploring the catalytic mechanism of the first dimeric Bcp: functional, structural and docking analyses of Bcp4 from *Sulfolobus solfataricus*. Biochimie 92, 1435–1444 10.1016/j.biochi.2010.07.006 20637256

[B18] Lohman J. R. Remington S. J. (2008). Development of a family of redox-sensitive green fluorescent protein indicators for use in relatively oxidizing subcellular environments. Biochemistry 47, 8678–8688 10.1021/bi800498g 18652491

[B19] Maeda K. Hagglund P. Bjornberg O. Winther J. R. Svensson B. (2010). Kinetic and thermodynamic properties of two barley thioredoxin h isozymes, HvTrxh1 and HvTrxh2. FEBS Lett. 584, 3376–3380 10.1016/j.febslet.2010.06.028 20594550

[B20] McKinney D. W. Buchanan B. B. Wolosiuk R. A. (1979). Association of a thioredoxin-like protein with chloroplast coupling factor (CF1). Biochem. Biophys. Res. Commun. 86, 1178–1184 10.1016/0006-291X(79)90241-9 219862

[B21] Meyer A. J. Brach T. Marty L. Kreye S. Rouhier N. Jacquot J. P. (2007). Redox-sensitive GFP in *Arabidopsis thaliana* is a quantitative biosensor for the redox potential of the cellular glutathione redox buffer. Plant J. 52, 973–986 10.1111/j.1365-313X.2007.03280.x 17892447

[B22] Montrichard F. Alkhalfioui F. Yano H. Vensel W. H. Hurkman W. J. Buchanan B. B. (2009). Thioredoxin targets in plants: the first 30 years. J. Proteomics 72, 452–474 10.1016/j.jprot.2008.12.002 19135183

[B23] Motohashi K. Kondoh A. Stumpp M. T. Hisabori T. (2001). Comprehensive survey of proteins targeted by chloroplast thioredoxin. Proc. Natl. Acad. Sci. U.S.A. 98, 11224–11229 10.1073/pnas.191282098 11553771 PMC58711

[B24] Nalin C. M. Mccarty R. E. (1984). Role of a disulfide bond in the γ subunit in activation of the ATPase of chloroplast coupling factor 1. J. Biol. Chem. 259, 7275–7280 6233280

[B25] Olry A. Boschi-Muller S. Branlant G. (2004). Kinetic characterization of the catalytic mechanism of methionine sulfoxide reductase B from Neisseria meningitidis. Biochemistry 43, 11616–11622 10.1021/bi049306z 15350148

[B26] Perez-Perez M. E. Martin-Figueroa E. Florencio F. J. (2009). Photosynthetic regulation of the cyanobacterium *Synechocystis sp*. PCC 6803 thioredoxin system and functional analysis of TrxB (Trx x) and TrxQ (Trx y) thioredoxins. Mol. Plant 2, 270–283 10.1093/mp/ssn070 19825613

[B27] Perkins A. Gretes M. C. Nelson K. J. Poole L. B. Karplus P. A. (2012). Mapping the active site helix-to-strand conversion of CxxxxC peroxiredoxin Q enzymes. Biochemistry 51, 7638–7650 10.1021/bi301017s 22928725 PMC3549014

[B28] Rouhier N. Gelhaye E. Gualberto J. M. Jordy M. N. de Fay E. Hirasawa M. (2004). Poplar peroxiredoxin Q. A thioredoxin-linked chloroplast antioxidant functional in pathogen defense. Plant Physiol. 134, 1027–1038 10.1104/pp.103.035865 14976238 PMC389925

[B29] Sarkar G. Sommer S. S. (1990). The megaprimer method of site-directed mutagenesis. Biotechniques 8, 404–407 2340178

[B30] Scheibe R. Anderson L. E. (1981). Dark modulation of NADP-dependent malate dehydrogenase and glucose-6-phosphate dehydrogenase in the chloroplast. Biochim. Biophys. Acta 636, 58–64 10.1016/0005-2728(81)90075-X 7284346

[B31] Stumpp M. T. Motohashi K. Hisabori T. (1999). Chloroplast thioredoxin mutants without active-site cysteines facilitate the reduction of the regulatory disulphide bridge on the g-subunit of chloroplast ATP synthase. Biochem. J. 341, 157–163 10.1042/0264-6021:3410157 10377257 PMC1220342

[B32] Tarrago L. Laugier E. Zaffagnini M. Marchand C. Le Marechal P. Rouhier N. (2009). Regeneration mechanisms of *Arabidopsis thaliana* methionine sulfoxide reductases B by glutaredoxins and thioredoxins. J. Biol. Chem. 284, 18963–18971 10.1074/jbc.M109.015487 19457862 PMC2707211

[B33] Yamazaki D. Motohashi K. Kasama T. Hara Y. Hisabori T. (2004). Target proteins of the cytosolic thioredoxins in *Arabidopsis thaliana*. Plant Cell Physiol. 45, 18–27 10.1093/pcp/pch019 14749482

[B34] Yano H. Wong J. H. Lee Y. M. Cho M. J. Buchanan B. B. (2001). A strategy for the identification of proteins targeted by thioredoxin. Proc. Natl. Acad. Sci. U.S.A. 98, 4794–4799 10.1073/pnas.071041998 11274350 PMC31913

